# Understanding digital health ecosystem from Australian citizens’ perspective: A scoping review

**DOI:** 10.1371/journal.pone.0260058

**Published:** 2021-11-15

**Authors:** Abraham Oshni Alvandi, Chris Bain, Frada Burstein

**Affiliations:** Faculty of Information Technology, Monash University, Clayton, Victoria, Australia; University of Notre Dame Australia, AUSTRALIA

## Abstract

**Background:**

Digital health (DH) and the benefits of related services are fairly well understood. However, it still is critical to map the digital health care landscape including the key elements that define it as an ecosystem. Particularly, knowing the perspectives of citizens on this digital transformation is an important angle to capture. In this review we aim to analyze the relevant studies to identify how DH is understood and experienced by Australian citizens and what they may require from DH platforms.

**Materials and methods:**

A scoping literature review was conducted across several electronic databases (ACM Digital Library, OVID, PubMed, Scopus, IEEE, Science Direct, SAGE), as well as grey literature. Additionally, citation mining was conducted to identify further relevant studies. Identified studies were subjected to eligibility criteria and the final set of articles was independently reviewed, analyzed, discussed and interpreted by three reviewers.

**Results:**

Of 3811 articles, 98 articles met the inclusion criteria with research-based articles–as opposed to review articles or white papers– comprising the largest proportion (72%) of the selected literature. The qualitative analysis of the literature revealed five key elements that capture the essence of the digital health ecosystem interventions from the viewpoint of the Australian citizens. The identified elements were “consumer/user”, “health care”, “technology”, “use and usability”, “data and information”. These elements were further found to be associated with 127 subcategories.

**Conclusions:**

This study is the first of its kind to analyze and synthesize the relevant literature on DH ecosystems from the citizens’ perspective. Through the lens of two research questions, this study defines the key components that were found crucial to understanding citizens’ experiences with DH. This understanding lays a strong foundation for designing and fostering DH ecosystem. The results provide a solid ground for empirical testing.

## Introduction

During the last decade, advances in information technologies have impacted the delivery of traditional health care services. Emerging devices and advanced health care systems have been embedded into a network to link the complex health care landscape as an ecosystem. Referencing the foundational work on technology ecosystems by Adomavicius, Bockstedt, Gupta and Kauffman [1, 2], we define a Digitally-enabled Health care Ecosystem (hereon termed “DH ecosystem”) as a network of health industry and technology components that influence each other’s operation, evolution and development while providing health care services. Components, in this context, include a human population whose members and characteristics change through health care actions, as well as information and transaction flows related to health care episodes [3, 4]. In this context, we differentiate DH ecosystems from DH platforms which constitute a set of technologies that facilitate digitally-enabled delivery of health care services. In a health care setting, it is advocated that a DH ecosystem should have, at its core, the intention of empowering its human population in achieving the health and wellbeing goals of the constituent individual citizens.

Recently, considerable research has been devoted to investigate technical and human issues which motivate the development and adoption of information technology (IT) in health services as part of progressing with a DH ecosystem. Specifically, numerous studies have explored the adoption of IT technologies by different user types, as well as the impact of IT interventions on health outcomes for individuals [5–9]. In their field study, Mercer et al. [10] demonstrated that older adults living with chronic illness would adopt wearable activity-tracking technologies as long as such technologies are perceived useful in promoting their self-awareness, improving their wellbeing and motivating physical activity.

There is research conducted from the viewpoints of health care professionals and policy makers to understand how consumers experience DH [3, 11–14]. For example, the study by Robertson, Walkom and Henry et al. [15] demonstrated that clinicians and DH consumers in Australia perceive the sustainability of the health care system differently [15]. The authors found that the increase in the cost of the health care services are of concern to patients, whereas funding-related issues linked to the ageing community and their access to the new technologies are more of concern to clinicians.

DH research has also focused on the privacy and security of DH platforms, among which the regulation and policy of health data governance have been subject to extensive discussion [16]. For instance, it has been debated who should take custody of the data even when the data collected are public or de-identified [17]. While patient consent should be obtained whenever possible to protect data, the mentioned concern has highlighted the importance of all DH stakeholders’ perspectives over the management of security and ownership of data. Wilkowska et al. [18] found that younger female users ascribed a higher tendency than males to safeguarding important consumer information, providing confidentiality on what is recorded or communicated via DH platforms, while also preferring that the design of devices that ensures non-stigmatizing services. In general, privacy has been identified as one of the main barriers for adoption of health care IT systems. A prime example of such an issue is personal health information recording systems such as My Health Record [19].

Despite this wide understanding of the importance of DH, and how the use of different technologies can provide solutions to optimize access to better and secure health care services, little is known about the citizens’ perspectives of the entire DH ecosystem [20]. It could be argued that the digital health sector holds its own operational views about what constitutes the reality in the current DH implementation from a professional and clinical stance. Yet, there is still little knowledge about the reality of the DH ecosystem from the holistic all-inclusive consumers’ perspectives. To the best of our knowledge, there is no clear description of the services as currently offered, perceived or desired by individual or community-based DH consumers in Australia [21, 22]. Thus, our study examines the nature, origin, limits of knowledge, beliefs and opinions of DH consumers in the context of the DH ecosystem. Fulfilling the vision outlined in the Digital Health Strategy for Australia [21] requires forming a good understanding of the defining factors of the Australian DH ecosystem and a variety of relevant citizen characteristics that form the social fabric of such a network. For example, the factors characterizing social groups may be defined by income level, ethnicity, education, languages and cultures, and linked to geographical factors. The DH Strategy implementation would also require to know what are the common diseases and conditions that have been addressed already by DH initiatives, or identify clear opportunities for future innovations for the benefit of citizens in those regions.

In order to bridge this knowledge gap, it was useful to review and map the relevant research terrain and identify contributing factors of digitally enabled health services, especially as they are experienced and perceived by “typical citizens” as stakeholders involved. This scoping review collated studies that explore DH services in the general Australian population. In this paper we aim to provide a macro view of the perspectives of Australian citizens on DH technologies through the review of current research and other relevant publications.

The rest of the paper is organized as follows: Section 2 explains the research method, the strategy and protocols of search. In this section, we identify the questions of the research and describe the procedure by which studies were identified. The methods employed to extract data and analyze the information are also explored in Section 2. Section 3 reports the results and defines the themes extracted from the reviewed studies, illustrating Australian citizens’ perception of the DH ecosystem. Section 4 discusses the findings; Section 5 identifies some limitations of this research; and Section 6 summarizes the conclusions.

## Methods

This scoping literature review was conducted by a research team at the [REMOVED FOR REVIEW] consisting of individuals with deep expertise in DH, health and social informatics. The methodology used in the research is outlined below.

### Search strategy

This review was conducted in accordance with the Preferred Reporting Items for Systematic reviews and Meta-Analyses extension for Scoping Reviews (PRISMA-ScR) guidelines [23, 24]. We followed the five key phases: (a) identifying the research question, (b) identifying relevant studies, (c) study selection, (d) charting the data, and (e) collating, summarizing, and reporting the results. Acknowledging the work of Arksey and O’Malley [25] and Pham et al. [26], we adopted the scoping framework in order to map the relevant DH literature using a wide range of research and non-research types of evidence. Due to the complex and heterogeneous nature of the domain of search, a scoping review was employed to bring out conceptual clarity to the specific research topics from diverse materials and perspectives. No meta-analysis was attempted accordingly.

### Research questions

In this study we set out to address the following key research questions via a structured literature review:

- What does the typical citizen in Australia (the “person in the street”) know about DH, if anything?
- What is the current experience with citizens’ requirements to DH?

### Identifying relevant studies

Studies were identified using a systematic searching method including pre-defined selection criteria. Our search of major library databases covered publication dates from 2014 to 2019 inclusive (a 5-year period).

#### Study eligibility criteria

The review material included clinical trials, as well as exploratory, field, experimental, systematic review, qualitative, and quantitative studies associated with the objectives of the review. The challenge was to paint a big-picture out of the literature found on the topic, so the inclusion process was not limited to studies of one type. Consequently, a review included research articles and white papers that cover the objectives of the study either directly or indirectly. The inclusion and exclusion criteria are presented in Table 1. As listed, the initial inclusion criteria were refined in a subsequent screening process to better represent the focus of the review.

**Table 1:** Selection criteria.

➢ Initial inclusion criteria:
• Articles published in the English language
• Articles published during 2014–2019 (i.e., last 5 years)
• Articles tagged with the term “Australia”
➢The screening process was guided by the additional inclusion criteria:
• Representing the perspective of consumer health informatics but not just clinicians’/providers’ attitudes
• Having Australian-oriented content
• Non-technical papers (e.g., description of an app or an algorithm development)
• Not discussing a direct clinical outcome
• Not being editorials and trial registration materials
• Professionally focused articles and opinions statements related to Australian DH
➢ During the final inclusion process, articles were included for the final review only if:
• Articles contributed to answers to the research questions of the study.
• Articles discuss Australian citizens’/consumers’ perspectives directly.

#### Target population–consumers

To classify the DH consumers in the identified dataset, we primarily referred to the definition of stakeholders suggested by Gagnon et al. [27]. They identified clinicians, decision makers, patients and the public as the major types of stakeholders. In addition, we consider healthy individuals or groups who may use digital tools and interventions to collect or access information on health-related issues and promote their wellbeing; equally we consider ill or injured people in the management of their health conditions. Thus, extending the concept of *consumer* to cover this group of stakeholders, we consider a “citizen” as the core stakeholder. Applying this definition of a consumer, we included any research that directly or indirectly provided insights into the citizen or consumer perspectives on this topic. Moving forward we further categorized stakeholders by means of a qualitative coding technique.

#### Target DH ecosystem technologies

Within the broad definition of the DH construct as the use of digital tools and interventions in health care and wellness, no restriction was made on the types of technologies included in the literature review. We argued that restricting the search to either one specific device or a range of tools could overlook the variety of the technologies that exist in the DH ecosystem.

#### Search protocols: Search terms

Based on our research questions, we formulated initial search keywords around three main themes, “citizen/consumer”, “Australia” and “digital health”. In the accessed bibliographic databases specified in the next section keyword searches were performed on title, abstract and keywords using Boolean operators “AND/OR”. The search syntax used is presented in Table 2 below. The search terms for the “citizen/consumer” theme included concepts of “citizen”, “consumer” and “patient”. For the “Digital Health” theme, the concept of “Digital Health” was used individually. In addition, the World Health Organization [28] defines the term digital health as an overarching term that includes various information and digital technologies. Therefore, the terms of “health informatics”, “mHealth”, “telemedicine”, “eHealth”, and “Electronic Health Records” (EHR) were included as synonyms, as these were identified as the technologies adopted in the baseline infrastructure for DH.

**Table 2:** Search syntax for literature search.

“(*Australia) AND (consumer OR patient OR citizen) AND ("digital health" OR "health informatics" OR "mhealth" OR "telemedicine" OR "eHealth" OR "EHR"*)”
With ***Search details that is*:** *("australia"[MeSH Terms] OR "australia"[All Fields]) AND (consumer[All Fields] OR ("patients"[MeSH Terms] OR "patients"[All Fields] OR "patient"[All Fields]) OR citizen[All Fields]) AND ("digital health"[All Fields] OR "health informatics"[All Fields] OR "mhealth"[All Fields] OR "telemedicine"[All Fields] OR "eHealth"[All Fields] OR "EHR"[All Fields]) AND ("2014/08/10"[PDat]*: *"2019/08/08"[PDat] AND "humans"[MeSH Terms])*]

#### Search protocols: Search sources

Online database literature searches were performed in August to November 2019 to obtain relevant articles to review. The electronic bibliographic databases used were: PubMed, Scopus, OVID, IEEE, ACM, Science Direct, SAGE, Mary Ann Linhert, and APO. Some additional papers were included due to the authors’ knowledge of the newer publications from the individual authors and local research groups working in the area.

To minimize the likelihood of missing relevant articles, we conducted a cited reference search for all the articles considered for the final review. Articles that were found this way were screened for duplication and for meeting inclusion criteria. We also used the Google Scholar search engine to identify all articles that have cited the original papers.

#### Data management

The search results were imported either as CSV files where direct export was provided from the searched libraries, or added manually to an Endnote database, then merged in Excel spreadsheet. Duplicates were identified in the Excel or Endnote and were removed accordingly.

### Study data set selection

**Fig 1:**
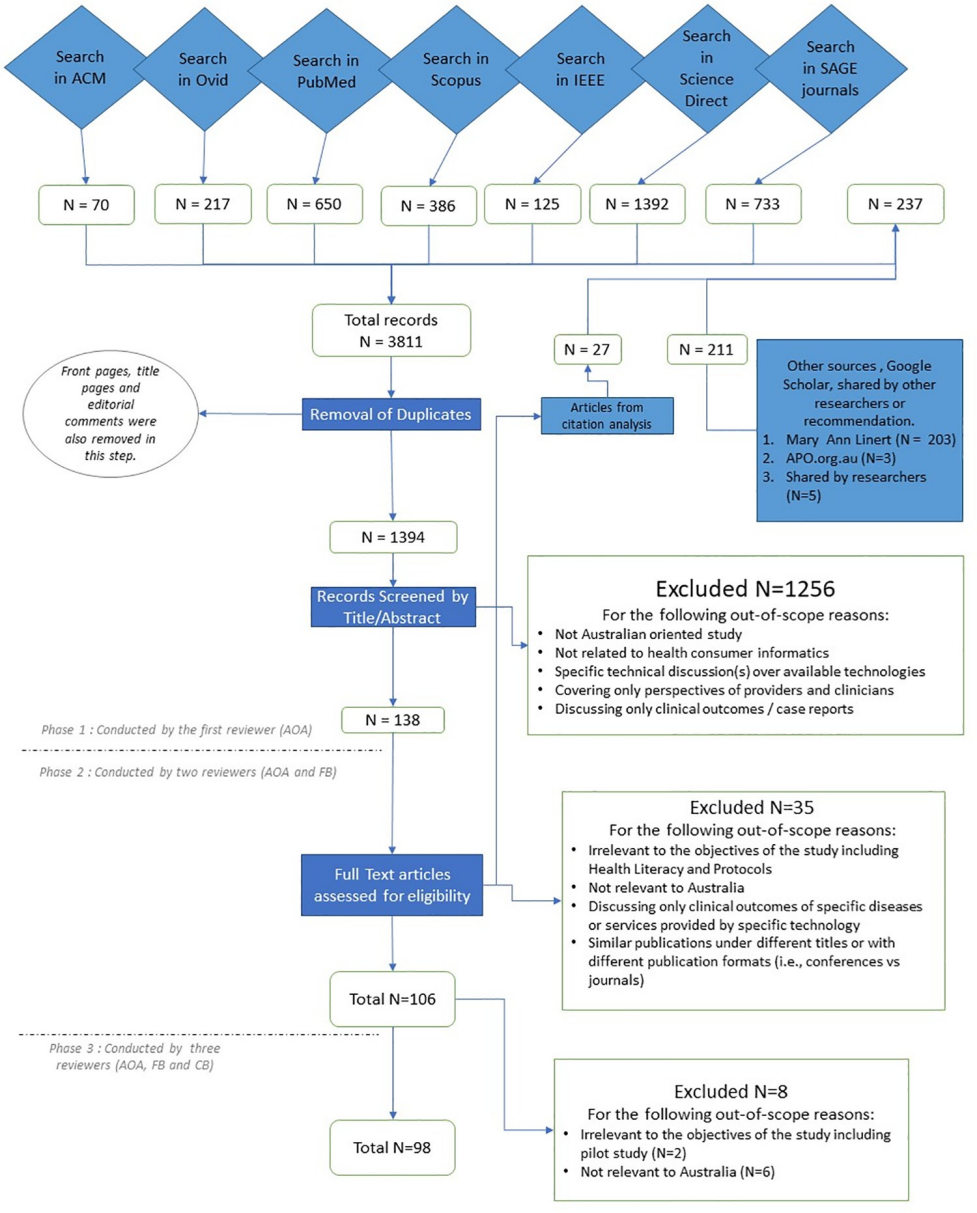
Flow chart of the study selection process.

As shown in Fig 1, the data set selection process was conducted in three phases. In the first phase, the first author (AOA) searched the databases which returned 3811 articles. Then, AOA applied the early screening process on the title, abstract and keywords of the articles. This abstract screening phase was based on the inclusion and exclusion criteria for the study. During this screening phase, the studies that specifically captured the perspectives of consumers and patients on DH (138 out of 1393) were selected.

During the second phase, papers were examined independently by two reviewers (the first and the third authors). In this phase, agreement between reviewers was assessed by calculating a Cohen’s kappa on their inter-coded decision points (i.e., inclusion and exclusion) [29]. A kappa value of 0.65 indicated a substantial agreement between both reviewers. The disagreements were discussed and resolved by both reviewers. This screening resulted in 106 articles.

The last phase included a full text review conducted independently by three reviewers (all three co-authors). This comprehensive evaluation yielded a final sample of 98 papers.

### Data extraction, synthesis and analysis

The final set of papers was considered from the point of view of the quality of the academic outlet. The SCImago Journal Rank (SJR) indicator was used as the primary source of the ranking of quality of these publications. Table 3 presents a summary of this quality of publications assessment.

**Table 3:** Quality of included journals and papers based on SJR.

	*Journals N(%)*	*Papers N(%)*
**Q1**	29(50)	37(37.37)
**Q2**	15(26)	35(35.35)
**Q3**	7(12)	18(18.18)
**Q4**	2(3)	4(4.04)
**Not Ranked**	5(9)	5(4.05)

During the screening phases, papers were coded in two dimensions. The first was regarding the reference details–specifically, authors, place of publication, year of publication, and type of study (i.e., theoretical discussion, experimental attempt, review, or white literature). The second dimension related to the level of relevance to the objectives of the study, methodology, and outcomes and recommendations for the papers to be included or excluded. During the analysis, each article was read in full and assessed by reviewers against these dimensions and relevance to the research questions. The personal evaluation of each reviewer was shared in a Google Drive spreadsheet template, a copy of which is found in S1 Appendix.

In the review process, if a new category or subcategory that had not been identified in the selected studies was discovered, it was allowed to be added to the list of themes and categories to capture further information. To validate the new category, researchers independently confirmed and compared the coding with other subsets of catalogued types.

### Study quality considerations

The quality of the included studies is not usually assessed in a scoping review [25, 30]. Due to the inclusion of all forms of research studies, i.e., review papers, perspectives such as reports and white articles, we acknowledge that the diversity in types of papers and various methodologies may present bias in locating, selecting, appraising and synthetizing studies. In order to offset this, reviewers inspected each other’s reasoning and decision making pertaining to papers marked for exclusion. Only where consensus was reached between all 3 reviewers were papers excluded at this last stage. For example, some studies written by Australian researchers as part of an international team do not specifically look at the Australia citizens’ perspective. Other studies might use the Australian cohort only as a minor proportion of the research cohort. Hence, we agreed to exclude such studies.

### Publication characteristics

Overall, after applying the inclusion and exclusion criteria we identified 98 articles for this scoping review (See S2 Appendix which summarizes the basic characteristics of the included articles). The articles were published in 58 journals and, according to SCImago Journal Rank (SJR), almost 50% of these journals were ranked of the highest tier (i.e., Q1) (see Table 3 and S3 Appendix regarding the SCIamgo Rank of each journal and the number of published papers).

Approximately 80% of the papers were published between 2016 to 2019, and research-based articles–as opposed to review articles or white papers for example–comprised the largest proportion (72%) of the selected literature.

**Fig 2:**
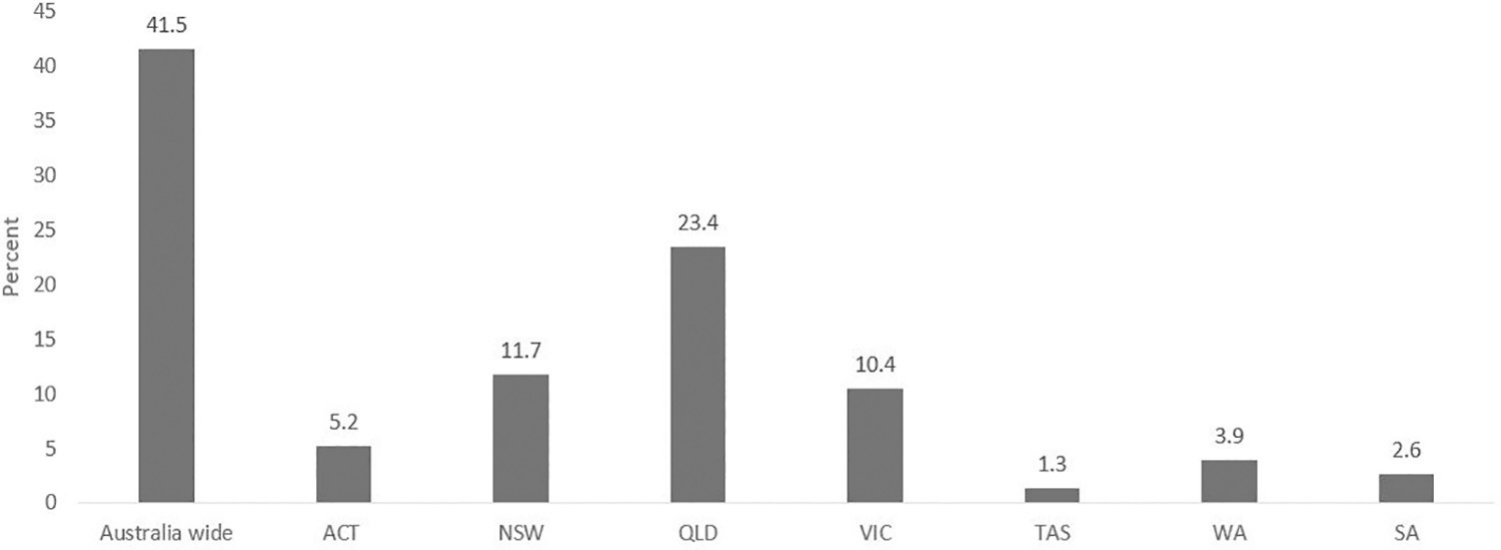
Distribution of research-based articles based on the location of studies.

Regarding the locations where research-based articles were associated, the experiments and trials were conducted mostly across Australia (41.5%), with Queensland as the main state of their focus. The states of Victoria and New South Wales were ranked in the next level with almost equal contribution (see Fig 2 for more details).

## Results and findings

The analysis of the literature revealed five overarching themes to describe how the DH ecosystem is understood and experienced by Australian citizens. In addition to the three themes set as the starting point for the literature search, i.e., “consumer and user”, “health care”, “technology”, the two main themes of “use and usability” and “data and information” were identified as the core foci of the reviewed literature. In the following subsections, we present the evidence derived in each of the study themes and classify papers according to the themes they cover. The complete set of references associated with the themes, categories and subcategories are listed in the relevant tables, while only a selection are used as illustrations in our discussion.

### Consumer and user

The first theme was derived from the analysis of the articles focusing on the stakeholders. The focus has been on consumers and users of the DH platforms and the community (as a whole) that these interventions were associated with. Unsurprisingly, consumers were strongly factored into all the retrieved research. As shown in Table 4, the stakeholders were classified into three categories.

**Table 4:** Summary of findings in the reviewed literature: Consumer and user.

*Category*	*Freq*.	*Refs*.	*Subcategories*	*Freq*.	*Refs*.
**Citizen/person**	2	[31, 32]	Professionals	6	[33–38]
			Patients	11	[33–35, 39–46]
			Individuals	8	[35, 36, 47–52]
**Public**			Community	2	[35, 53]
			People	1	[54]
			Culture	9	[33, 48, 50, 53, 55–59]
			Occupation	1	[39]
			Income	2	[49, 53]
			Education	6	[49, 56, 60–63]
			Groups	4	[49, 55, 64, 65]
			Old people	12	[31, 39, 47, 49, 51, 59, 61, 66–70]
			Aging population	9	[41, 49, 55, 60, 62, 67, 71–73]
			Young people	9	[50, 60, 64, 69, 74–78]
			Adults	4	[49, 64, 71, 79]
			Gender	4	[60–62, 66]
			a. Male	2	[49, 63]
			b. Female	10	[49, 56, 63, 67, 75, 80–84]
			Family	5	[33, 39, 51, 67, 85]
			Children	5	[38, 40, 59, 86, 87]
			Parents	2	[59, 86]
			Vulnerable	1	[35]
			Indigenous	5	[40, 53, 59, 88, 89]
			Veterans	1	[59]
			Students	3	[60, 64, 90]
**Government**			Organizations	6	[35, 42, 50, 91–93]

As the basic category, consumers were specifically characterized in the literature by the concepts of *citizens/person* [31, 32]. Eleven of the reviewed studies discussed citizens/persons as *patients* who demand and consume DH platforms for many reasons [33–35, 39–46]. Although patients were recognized as the main recipient of DH services, non-patient *individuals* were also another significant subcategory that were specified in the eight studies. Healthy individuals, in particular, were discussed significant group in which individuals were users of wellness programs [52], carers for patients with long-term conditions [47, 51], and administrative staff from health care organizations [48, 50]. Medical *professionals* were also considered consumers who are affected by and interact with the DH platforms as part of their professional practice [33–38].

At the next level down, the stakeholders were categorized by *public* groups [48, 49, 59], and *governmental* bodies [42, 92, 93]. These categories were characterized by a great degree of diversity. As listed in Table 4, 19 subcategories defined how stakeholders are represented within the public, among which aging people [67], students [64, 90], family [51], and children [86, 87] were the main targeted consumer demographics. Despite their importance, there was little evidence to support some subcategories. There was no strong evidence related to a special group of Australian society such as serving and ex-serving veterans [59] or consumers grouped by particular community characteristics such as occupation [39].

Nine studies highlighted the importance of culturally and linguistically diverse (CALD) background as a directing factor in the use and design of DH technologies. In spite of the importance of CALD, only two studies have provided experimental figures on how CALD people experience DH technologies [48, 56]. CALD people are not homogenous but only one article has mentioned participants with Anglo-Celtic and Asian ancestry [56] and one study has discussed LGBTIQ people expressing that these intended audiences confront discrimination and fear to use DH technologies (i.e., My Health Record) [54].

### Technology

The second theme comprised studies focusing on the apparatus used within Australia’s DH ecosystem. According to Table 5, a variety of *health information systems* and *devices* were mentioned in the reviewed studies.

**Table 5:** Summary of findings in the reviewed literature: Technology.

*Category*	*Subcategories*	*Freq*.	*Refs*.
**Systems**	Online	14	[39, 56, 60, 62, 63, 70, 71, 74, 80, 81, 91, 94–96]
	Telemedicine	6	[37, 40, 55, 88, 97, 98]
	a. Wearables	7	[31, 78, 82, 84, 99–101]
	b. Apps	18	[37, 44, 50–52, 76–79, 82, 84, 86, 99, 100, 102–105]
	c. Video conferencing	10	[39, 40, 46, 57, 67, 68, 76, 87, 106, 107]
	Internet	16	[32, 44, 47, 52, 58, 61, 62, 68–70, 81, 85, 86, 96, 106, 108]
	a. m-health	14	[32, 34, 52, 60, 64, 69, 70, 81, 91, 97, 99, 101, 102, 109]
	1. MDM Services*	1	[38]
	b. e-health	21	[36, 42–44, 49, 52, 57, 60, 61, 63, 70, 71, 73–75, 80, 90, 92, 108, 110, 111]
	c. PCEHR**	10	[36, 41–43, 49, 57, 92, 95, 112, 113]
	d. EMR***	5	[35, 43, 45, 49, 113]
	e. My Health Record	8	[35, 47, 48, 56, 59, 92, 112, 114]
	f. Websites	9	[47, 61, 62, 74, 75, 78, 83, 96, 110]
	1. Portals	1	[45]
	g. Social Media	10	[32, 50, 56, 61, 64, 75, 78, 83, 91, 96]
	Telehealth	16	[33, 37, 38, 46, 66–69, 72, 85, 89, 107, 115–117]
	Artificial Intelligence / Sensors/ Analytics	1	[118]
**Devices**	Media	3	[36, 70, 78]
	Phones	9	[55, 61, 68, 69, 86, 91, 106, 111, 119]
	a. Text/SMS	4	[52, 61, 65, 75]
	Smartphones	15	[34, 37, 44, 51, 60, 64, 75–77, 84, 96, 99, 102, 105]

*MDM: Mobile Device Management Services.

**PCEHR: The Personally Controlled Electronic Health Record.

*** EMR: Electronic Medical Records.

The most commonly referenced systems were *online services*, *telemedicine* including *wearables* and *video conferencing* platforms, *internet-based* interventions including *e-health*, *m-health and electronic health records*. In particular, 16 articles addressed *telehealth* as the most widely used and acknowledged system among the consumers. Telehealth was employed in the studies to provide various remote health care services including dentistry [37], paediatric cerebral palsy [38], kidney disease [69], and chronic conditions [107, 115].

Regarding the equipment, *phones* including *smartphones* were the major devices that were employed or mentioned in the studies. This is explainable as these devices supported the infrastructure for accessing health care *apps*.

### Health care

Health care was the third major theme related to the aspects of the DH ecosystem. The review resulted in eight major categories that define DH as a means to provide, maintain and improve health care. As illustrated in Table 6, it was found that digitally enabled health care is exercised in several studies for enabling *self-care*, *supporting and helping* users regarding their *diseases and wellbeing*, and for clinical *decision making* including treatments, outcomes, and prescription of medications. There also was a contribution of *education and health literacy* on the functioning scope of DH. DH platforms were used to educate consumers and enhance the awareness about patients’ health conditions [51, 68]. The final category is *care providers* which consists of three subcategories. Eighteen studies demonstrated that the use of DH was situated in all services that are provided at clinics and hospital settings and at both private and public sectors of Australia. The special subcategory identified in five studies was supporting administrate tasks, such as booking appointments and processing admissions.

**Table 6:** Summary of findings in the reviewed literature: Health care.

*Category*	*Freq*.	*Refs*.	*Subcategories*	*Freq*.	*Refs*.
**Self-care**	10	[52, 61, 62, 67, 70, 76, 84, 97, 99, 100]			
**Well-being**	12	[41, 47, 50, 52, 55, 64, 67, 78, 79, 82, 101, 110]			
**Care Provider**			Clinics	6	[53, 66, 71, 73, 111, 120]
			Hospitals	7	[42, 43, 66, 73, 76, 94, 95]
			Appointments/admission	5	[31, 43, 53, 95, 111]
**Education / health literacy**	16	[34, 38, 39, 41, 50, 52, 53, 58, 59, 62, 63, 70, 74, 95, 103, 121]			
**Help**	2	[74, 119]			
**Support**	14	[42, 53, 55, 74, 76, 78, 80, 81, 83, 94, 102, 103, 115, 119]			
**Diseases**			Cancer	3	[81, 97, 110]
			Dietary/Nutrition	1	[75]
			Diabetics	10	[52, 53, 55, 76, 79, 89, 95, 103, 105, 109]
			Chronic	10	[35, 41, 52, 55, 59, 62, 67, 69, 72, 73]
			Heart	1	[39]
			Skin	4	[40, 77, 97, 120]
			Rehabilitation	5	[31, 39, 66, 94, 122]
			Blood	1	[52]
			Mental Disorders	11	[47, 57, 59, 61, 65, 71, 74, 84, 85, 96, 108]
			Maternity	1	[43]
			Dentistry	1	[37]
			Asthma	2	[52, 70]
			Injury	1	[74]
			Addiction	2	[80, 85]
			Communication disability	1	[47]
			Irritable bowel syndrome (IBS)	1	[44]
			Cerebral palsy (CP)	1	[38]
			Dementias	2	[51, 61]
			Musculoskeletal conditions / Osteoarthritis	2	[106, 107]
**Decision making**	14	[37, 40–42, 58, 63, 66, 70, 90, 91, 95, 103, 110, 121]	Treatments	11	[35, 41, 42, 58, 61, 71, 75, 81, 99, 106, 121]
			Medications	3	[49, 55, 102]
			Results/outcomes	8	[39, 41, 49, 58, 67, 99, 104, 115]

Under the key category of diseases, 19 types of illnesses have been addressed in the literature describing DH initiatives in Australia. All of the stated diseases were mentioned in the studies in three different ways: 1) there were general health issues that were described in the participants during the research; 2) there were health conditions of users of technology (e.g., cancer) that were targeted for the DH research purposes [110]; or 3) diseases were targeted as a key health issue of the DH intervention that was meant to be evaluated by the relevant consumers [105]. Regarding frequency, mental disorders, chronic health problems and diabetes were mentioned more than other diseases in the studies.

### Use and usability

Several factors emerged relating to the fourth major theme that addressed how consumers can use or experience technologies and how that helps to achieve a health goal effectively, efficiently and satisfactorily (See Table 7).

**Table 7:** Summary of findings in the reviewed literature: Use and usability.

*Category*	*Freq*.	*Refs*.	*Subcategories*	*Freq*.	*Refs*.
**Usability**					
**Acceptability**	27	[34, 36, 37, 39, 42, 52, 53, 56, 62, 65, 75–77, 79, 81, 90, 92, 94–97, 108, 110–112, 115, 122]			
**Perception**	27	[31, 34, 36, 37, 39, 41–43, 52, 58, 61, 73, 76, 77, 79, 88, 90–92, 94, 95, 97, 103, 110, 111, 115, 121]			
**Effectiveness**	10	[34, 53, 58, 60, 74, 75, 77, 79, 92, 102]	Efficacy	8	[36, 40, 52, 64, 67, 79, 94, 115]
**Satisfaction**	16	[32, 40, 45, 46, 58, 65, 77, 81, 88, 90, 97, 100, 109, 119, 120, 122]			
**Accessibility**	27	[34, 36, 37, 39, 40, 42, 44, 47, 48, 52, 53, 61, 66, 71, 75, 78–80, 90, 92, 94, 96, 111–113, 115, 122]			
**Trackability**	6	[55, 78, 84, 99, 100, 103]			
**Adaptability**	11	[31, 41, 52, 53, 57, 72, 77, 81, 103, 111, 112]			
**Ease of use**	18	[36, 37, 39, 43, 52, 66, 73, 77, 81, 82, 92, 94, 97, 103, 106, 107, 111, 115]			
**Communication**	15	[34, 43, 45, 48, 59, 64, 76, 85, 89, 92, 95, 99, 109, 111, 121]			
**Engagement**	15	[35, 36, 39, 41, 45, 50, 52, 56, 57, 65, 76, 82, 84, 94, 107]	Personalization	4	[78, 82, 102, 123]
			Gamification	1	[52]
			Customization	3	[52, 78, 103]
			Collaboration/peer support	2	[44, 70]
**Use**					
**Barriers**	25	[33, 39, 40, 45, 51, 53, 56–59, 70, 76, 77, 80, 81, 84, 92, 94, 100, 101, 107, 108, 112, 115, 121]			
**Relationship**	6	[35, 40, 59, 78, 100, 102]	Affect and emotion	19	[39, 41, 51, 56, 61, 72, 76–79, 83, 84, 88, 94, 97, 100, 102, 121, 124]
			Connectedness	6	[44, 56, 78, 83, 84, 102]
**Limits**	1	[82]			
**Dependency**	1	[41]			
**Tech Literacy**	15	[39, 48, 50, 51, 56, 59, 62, 68, 70, 73, 89, 92, 107, 111, 121]			
**Evaluation**	1	[51]			
**Training**	9	[38, 39, 47, 50, 51, 53, 55, 58, 78]			
**Functionality**	14	[37, 49, 52, 55, 56, 61, 76, 77, 81, 92, 94, 95, 103, 115]	Performativity	7	[41, 42, 66, 92, 95, 97, 115]
**Facilitation**	1	[32]			
**Interventions**	12	[47, 51, 58, 60–62, 74, 79, 81, 94, 95, 98]			
**Screening**	5	[40, 61, 81, 95, 120]			
**Monitoring**	14	[39, 44, 51, 52, 55, 75, 76, 81, 82, 94, 99, 100, 115, 125]			
**Manageability**	23	[35, 40–42, 46, 47, 49, 52, 53, 55, 61, 62, 67, 69, 79, 90, 92, 94, 102–104, 110, 121]			
**Development**	5	[35, 48, 59, 75, 81]	Design	14	[41, 48, 51, 52, 55, 61, 76, 77, 81, 84, 90, 91, 115, 120]
			Aesthetics	1	[55]
**Deliverability**	11	[33, 42, 46–48, 53, 55, 60, 66, 90, 94]	Accuracy	11	[31, 37, 40, 59, 77, 78, 82, 84, 95, 97, 110]
			Feasibility	10	[40, 51–53, 61, 65, 102, 110, 120]
**Attitude**	10	[38, 48, 56, 59, 66, 71, 77, 90, 97, 112]			
**Experience**	15	[31, 36, 41, 46, 51, 55, 56, 66, 67, 71, 106, 111, 115, 120, 126]			
**Adherence**	2	[85, 109]			

The analysis provided strong evidence that the authors differentiate *use* of technology and its *usability* when it comes to explaining the needs and expectations of DH consumers.

Ten major categories were batched together regarding the ***usability*** of DH technologies as experienced by the Australian population. As listed in Table 7, *user perception*, *accessibility*, *acceptability* and *ease of use* were the most commonly mentioned factors. Under the usability segment, health consumers identified s*atisfaction* and *adoption* of health technologies as very important design factors. Other key factors in usability of DH technologies were *communication* and *engagement*.

Regarding *engagement*, four indicators were suggested in the literature to increase the involvement of consumers with DH health care services. Firstly, DH platforms should provide a *personalized strategy* that involves contextualizing the better experience for user and customer satisfaction [78, 82, 102, 123]. Secondly, it has been proposed that DH products should be *customizable*. That said, consumers should be empowered to be involved in the design of the products or services and DH products should be adopted to achieve the personalization strategy for individuals or groups [52, 78, 103]. Thirdly, the incentivization of consumers’ engagement and activities will be increased by the *collaboration* between stakeholders or by receiving support from peers [44, 70]. The final indicator was implementation of game—style mechanisms also known as gamification [52]. The gamification was discussed as a leveraging vehicle that can enhance consumers’ tendencies to engage with DH health care services.

Eighteen other categories were synthesized to define factors related to the ***use*** of DH technologies. Among the enhancing factors of the intention to use DH services, the literature highlighted the *functionality* of DH platforms, the *deliverability* of accurate services, *training* programs, and the *relationship* between consumers and providers. In addition, it became evident from the literature that citizens may best manage DH with a positive *attitude* towards the use of technology, and prior *experience* with computers or other digital technologies. The potential to boost *adherence* to DH services was also linked to technological *performance*, *manageability and feasibility* of systems and services, proper health care product *design* and the *aesthetic* value of the products. It was also found that DH consumers want these products developed with functionality, enabling screening and monitoring of their health status.

On the other hand, several *barriers and limitations* were listed as adversely impacting the use of DH platforms. More generally, it has been discussed that there will be no intention to use DH services if products do not provide *practical assistance* or do not ensure *safe access* to services [53, 116]. As part of the barriers, it also has been noted that *personal characteristics* of the consumers, such as cognitive constraints and anxiety in using technology, can impede the adoption and acceptance of DH.

Use and usability are a dyad that were mentioned in the literature mainly in association with the ***needs*, *preferences and requirements*** from DH platforms (See Table 8). Of the major categories identified, literature suggests that DH consumers strive to be *empowere*d in terms of using and *controlling* their health-related tasks and information. More importantly, consumers wish to have DH as an assisting medium in *preventing* diseases or increasing their *knowledge* about DH services. *Affordability* of services, and experiencing the *benefits* of and *value* from DH were other factors highlighted in increasing citizen usage of DH.

**Table 8:** Summary of findings in the reviewed literature: Needs, preferences and requirements.

*Category*	*Freq*.	*Refs*.	*Subcategories*	*Freq*.	*Refs*.
**Control**	16	[31, 36, 42, 45, 48, 49, 59, 67, 71, 78, 83, 84, 90, 100, 102, 113]			
**Empowerment**	9	[35, 41, 42, 49, 58, 67, 83, 90, 113]			
**Prevention**	2	[58, 121]			
**Care services**	1	[93]	Consistency	1	[58]
			Timing	14	[31, 39, 76, 77, 80, 81, 83, 84, 94, 106, 111, 120, 121]
			Responsiveness	1	[102]
			Efficiency	4	[42, 43, 58, 106]
			Motivation	11	[39, 41, 52, 61, 67, 83, 84, 102, 115, 121]
			Improvement	11	[31, 39, 41, 43, 48, 49, 58, 67, 82, 92, 121]
			Involvement	5	[48, 56, 58, 84, 118]
			Integrity	5	[59, 82, 95, 103, 110]
			a. Interoperability	7	[52, 59, 73, 82, 84, 95, 121]
			Quality	9	[31, 37, 52, 58, 63, 92, 95, 101, 103]
			Usage/utility	18	[35, 37, 41, 43, 49, 59, 61, 64, 69, 71, 72, 79, 83, 84, 90, 103, 111, 120]
			Availability	11	[31, 38, 40, 42, 53, 73, 76, 80, 94, 95, 111]
			Mobility	4	[38, 72, 88, 121]
			Benefits	13	[40, 45, 48, 52, 56, 70, 71, 73, 76, 85, 92, 109, 121]
			a. Cost/affordability	22	[31, 38, 40, 42, 45, 56, 61, 66, 73, 76–78, 81–84, 91, 95, 106, 115, 121, 127]
			b. Value	10	[36, 41, 43, 48, 52, 58, 61, 66, 78, 111]
			Sustainability	2	[50, 53]
			Accountability	3	[90, 95, 123]
**Awareness**	14	[31, 36, 48, 52, 56, 57, 59, 60, 81, 83, 92, 100, 112, 121]			
**Participation**	2	[51, 92]	Location	2	[40, 51]
			Regions	10	[62, 67, 69, 73, 76, 80, 92, 106, 111, 120]
			Rural/remote	19	[33, 35, 37, 38, 40, 46, 53, 57, 59, 62, 66, 69, 73, 76, 80, 87, 88, 97, 106]
			Urban	7	[38, 59, 62, 76, 80, 106, 120]
			Home	6	[31, 55, 61, 66, 72, 97]

The location of consumers was found to be a noticeable factor in the adoption and employment of DH technologies. Rural and remote areas have been spotlighted in particular, reporting high trends of DH technology consumption. This is due to multiple factors including poorer access to and use of health services, distance to health services, low levels of education and employment issues. As indicated above (Fig 2), the review highlighted that one Australian state, Queensland, had the most innovations implemented and tested.

### Data and information

The last major theme identified was *Data and Information*. Bellow, Table 9 presents data and information-related factors that were identified in the selected articles associated with DH interventions. In the vast majority of the papers, access to up-to-date health data, and confidential and comprehensive wellbeing information were considered as vital by consumers. Trust in services and in the handling of health data were also featured in relation to the privacy concerns of citizens.

**Table 9:** Summary of findings in the reviewed literature: Data and information.

*Category*	*Subcategories*	*Freq*.	*Refs*.
**Data and Information**	Privacy	24	[31, 32, 36, 40, 47, 48, 50, 52, 56, 59, 66, 77, 78, 80, 81, 90, 92, 94, 99, 103, 106, 108, 113, 121]
	a. Identifiability	4	[43, 48, 49, 102]
	b. General Data Protection Regulation (GDPR)	1	[113]
	Policy	11	[31, 35, 40, 47, 48, 58, 59, 76, 90, 95, 113]
	a. Standardization	4	[95, 97, 113, 121]
	b. Sustainability	1	[58]
	Ethics	6	[31, 41, 47, 50, 56, 99]
	Security	14	[31, 36, 43, 44, 47, 48, 52, 56, 59, 78, 92, 102, 108, 121]
	a. Risk	5	[31, 36, 70, 76, 77]
	b. Concerns	9	[31, 49, 56, 59, 77, 78, 99, 104, 121]
	c. Confidentiality	10	[31, 36, 40, 44, 66, 77, 90, 91, 108, 115]
	d. Safety	5	[31, 39, 58, 95, 104]
	Strategy	6	[50, 61, 71, 91, 113, 121]
	Content	2	[50, 52]
	a. Source	9	[34, 38, 52, 78, 84, 91, 92, 103, 121]
	b. Authorization	4	[31, 99, 103, 113]
	c. Authentication	3	[31, 104, 113]
	d. Consent	5	[31, 48, 49, 99, 113]
	e. Ownership	4	[31, 33, 48, 78]
	f. Shareability	11	[31, 42, 46, 48, 50, 52, 90, 92, 95, 101, 115]
	g. Trust/Reliability	19	[36, 48, 52, 56, 59, 61, 70, 77, 78, 83, 90–92, 95, 97, 102, 117, 121, 128]

At the governance level, legal concerns and ethical considerations were highlighted, including issues such as consent and authentication for accessing data when developing remote monitoring or self-tracking systems.

## Discussion

In this section, we present the key findings from the literature review to address the research questions, followed by the limitations of the study.

We began this research with the goal of answering two primary research questions. We wanted to discover through analysis of the relevant literature: 1) What does the typical citizen in Australia (the “person in the street”) know about DH, if anything? And 2) What is the current knowledge about citizens requirements of DH? (i.e., what are their needs, experiences and desires?)

In terms of answering **the first research question,** there was a lack of evidence on how the different stakeholders including patients understand DH platforms and how such platforms work. At the same time, the review led to several interesting insights that are derived from what the analysis revealed indirectly. Many of the issues (e.g., citizen connection and empowerment, technologies and benefits of DH to citizens) are captured in a number of the dimensions derived on the basis of the thematic analysis, including the “Consumer/user” and “Use and usability” themes. For example, numerous papers indicated a good level of awareness of DH as evidenced through current, or intended, usage by citizens of classical DH tools such as apps [52, 79] or via their involvement in digitally-enabled health care models like cardiac tele-rehabilitation [39, 94, 122]. The importance of DH awareness was further evidenced by Nicholas et al. [126] who examined people with bipolar disorder. They found that 94% of young respondents (aged 18–30) reported daily smartphone use, while only 40% were using apps to help in their own self-management. It could be argued therefore that even in this young population with a clear need for optimal health care, usage of specific apps for this purpose was quite low.

In rural Australia, awareness of digital health care platforms is substantial. For instance, in a random survey of nearly 400 adults in South East Queensland, nearly 80% of them already had access to telehealth services [73]. The selection bias could clearly affect this result but Queensland seems to be ‘home’ to the majority of the empirical studies in DH in our sample. It is still an encouraging fact to take as an evidence in addressing the first research question—that such a level of awareness and engagement around DH exists in some parts of rural and regional Australia. Compared to the rural areas, no specific studies were found to draw the pattern of embracing telehealth services among urban consumers. However, the recent Medicare data [129] demonstrates that awareness of telehealth availability is steadily increasing in urban areas as this platform can facilitate healthcare for metropolitan consumers by helping remove geographical restrictions, accelerate care and provide more cost-effective services.

With regard to DH platforms, several devices and information systems were reported in the reviewed studies, with apps being the predominantly assessed tools. These findings align with recent trends in Australian consumers’ perceptions of their use of DH technologies such as apps [130]. However, there are some counterpoints to that overall picture. It is evident from the reviewed literature that Australian consumers are unaware of the various types of DH technology or opportunities available. For example, in one study, it was found that there is little awareness about existing technologies such as My Health Record in a sample of Australian female users [56]. Zhang et al. [111] highlight an important point regarding the dynamic and diffusive nature of technology (in their case, an e-appointment system) and its subsequent uptake by users over time. Thus, awareness of DH by ordinary citizens needs to be framed taking into consideration this current reality, as well as their future changing requirements.

Overall, the results of the review provide a strong indication that Australian citizens are willing to receive and ready to employ DH services. This is based on the perception that the effectiveness of platforms, or health-related outcomes and affordability of DH is associated with cost-effective or cost-saving processes. However, we did not find any specific report on economic data to use as evidence of the direct impact of DH interventions on health care ecosystems. There is no data available on the rates of increase in the health level or life expectancy of consumers, the reduction in outpatient-inpatient presentations, or decrease in cost and frequency of health care services due to the deployment of DH innovations. Additional research is needed to identify the impact of DH ecosystems on all key stakeholders (i.e., patient, payer, provider, and organization) compared to traditional health care services.

With regard to **the second research question** on the needs and desires of Australian citizens when it comes to DH tools and interventions, the literature review captured some key information.

One important dimension that came up in the literature is the need for consumers to have trust in how their sensitive health and wellness data is stored and managed in the DH ecosystem. This issue is already considered to be of concern for DH ecosystem implementation, but our review identified some empirical evidence based on research studies conducted in Australia. An example includes the research on a relevant ethical framework conducted by Segura Anaya et al. [31]. This issue was also raised in several papers concerning the implementation of the Australian national My Health Record systems (formerly called the PCEHR)–for example, the study by Jang-Jaccard et al. [33]. These and associated issues are collectively well captured within the “Data and Information” theme of DH ecology identified as one of the major themes as an outcome of this review.

A study by Lupton [78] with a small group of young Australians (n = 30 and 16–25 years) provided valuable insights into what this group are getting, and expected to get from DH tools and interventions. The author highlighted that the “opportunities for connection, emotional support, interacting with and listening to others were the vibrancies that animated the participants’ enactments of seeking and finding health information and support, that kept them googling, reading the content of websites, social media platforms and online forums, watching videos and using apps and wearable devices" (p.11).

It is important to build a DH platform or new application based on the wants and needs of the customers [55, 103]. This consumer centric process should be constantly monitored from the start and throughout the use of digital health technologies. Evidently also, it appears that DH tools and intervention programs should consider user age as an important factor in their design. It was observed, for example, that the use of technologies for health-related purposes can decrease as citizens age [49], and that older generations may prefer to access technology-based services via non-mobile devices such as PCs or laptops [60].

Another piece of research by Trawley et al. [79] provided useful insight into the key issues of perceived and actual relevance and utility when it comes to DH tools and interventions for patients with specific conditions. In their study regarding apps to support diabetic patients, they found that of the 76% (n = 607) of their respondents were not using apps, the most commonly reported reason was a belief by patients that apps would not help with the management of their diabetes (42%). The authors went on to state that “although the number of diabetes apps available on the market is increasing exponentially, they are not meeting the needs of people with diabetes. Relatedly, the most popular apps used for diabetes self-management were not diabetes-specific. More research is needed into understanding how the needs of people with diabetes could be supported by an app” (p.736). This observation may also inspire future research to shed some light on what are the critical factors to ensure sustainability of usage of other health and wellbeing DH tools.

A study by Anderson et al. [52], although based on a relatively small sample (n = 22), provided some insights about user desires for DH tools based on the user narratives they elicited. For instance, they found potential benefits of apps as a memory aid for performing blood sugar checks in diabetic patients, sharing self-management tips with other migraine sufferers, and also about the potential of more interconnectivity of raw data from multiple apps then aggregated into a single view.

Finally, the culturally and linguistically diverse backgrounds, gender, age, and socio-economic status of consumers were recognized as enablers or barriers for DH ecosystems. For example, there is a lack of research particularly related to the use, perception, and delivery of DH technologies among Aboriginal and Torres Strait Islander people. Within the period of search, we discovered only four studies that had directly addressed the use of DH among Australia’s Indigenous population. Those studies are related to delivering diabetes care (tele-diabetes) and acceptability of telemedicine [40, 53, 88, 89].

### Limitation and future research

This review was restricted to the published literature since 2019. We excluded studies published since 2020 due to the impact of the coronavirus pandemic. Evidently, the current worldwide outbreak of COVID-19 has been a catalyst to accelerate the use of DH platforms, and specifically telehealth [131, 132]. However, we believe this unprecedented period has triggered adoption of digitally enabled health care in specific ways. The adoption was impacted by the unusual pressures on health care under restricted resources, lack of completed evidence-based studies and temporary lockdowns, which should be studied independently.

Given the systematic and inclusive nature of our approach, this review aimed to establish a contextual understanding of consumers’ perspectives rather than generalizable conclusions. The interpretation of small scaled experimental studies was considered carefully. We were cautious not to deduce knowledge from pilot results related to issues such as the effectiveness of the DH interventions on consumers’ health status. We included a few comparative studies between Australia and other countries such as India; however, our findings were only focused on the DH status in Australia. Hence, it would be interesting if treated as a benchmark to explore the issues of DH ecosystem components in other countries.

Our study has highlighted a number of areas for future research. For instance, studies may further evaluate citizens’ appropriation of the DH ecosystem in the Australian context. In particular, a very significant area of research lies in better understanding the needs and perceptions of those who live in regional and remote areas, as well as those who are underprivileged such as migrants, culturally and linguistically diverse citizens, and those living with special health conditions. It is noteworthy that studies within the DH context have not examined specific categories of diseases (e.g., dermatological conditions) within Indigenous Australian patients (see: [40]). Also, there is insufficient knowledge about what the DH adoption status is among immigrants with Cultural and Linguistic Diversity (CALD).

Regarding the usage of DH platforms, future research should amplify the impact of technological interventions on consumers’ health outcomes by incorporating gamification which is a hot topic internationally, and could be an area that requires supplementary research in the Australian context (see: [52]).

In this study, we did not seek to explore the design and implementation of digitally enabled health services as components of that DH ecosystem. Our intention, in terms of continuing research, is to go on to answer the question, what does a supportive DH ecosystem need to look like to satisfy the needs and desires of citizens when it comes to DH? In subsequent research we will more explicitly investigate how the dimensions identified in the current review can assist in forming a DH ontology to help in understanding the scope and structure of the DH ecosystem in Australia. Such understanding can in turn be used to guide the continued evolution of a sustainable and useful DH ecosystem.

## Conclusions

This scoping review provides an overview of how Australian citizens understand and experience DH and also identify what they require from DH platforms. The review examined the literature related to DH and health care research across five years (2014–2019). The results suggest that the available evidence for presenting a clear and succinct description of a DH ecosystem from the perspective of Australian citizens is currently fragmented and lacks maturity in general understanding of its dimensions. A literature review showed that most of the current studies have specifically defined conclusions and were oriented to investigate the practice of introducing or using specific technologies for specific groups of consumers in some explicit context. The findings, however, suggest that more research is required to fully understand the global perspective on DH ecology in Australia both as it stands, and as it needs to be extended, to optimally support its citizens. For these purposes, the themes identified in our review and the relationships between them provide a solid foundation towards creating such a global vision.

It is essential to learn more about how technologies or platforms are used and perceived by different citizen groups. This comes with an additional step to understand how the efficacy of, and trust in DH tools and interventions can be facilitated by looking at common policies and procedures which were studied so far. Importantly, there is also a need to further assess if and how quality health care services are delivered to Australian citizens through DH–however, this is an exercise in ongoing vigilance.

Last but not least, the advantage of considering health care provision via digital platforms as part of an optimal ecosystem is that such a perspective is focused on empowering consumers. Although it is not easy to indicate exactly what should drive the shift from passive to empowered customers, the key element is associated with provision of full control of their health and wellbeing (activation) and facilitating them to remain connected within that ecosystem (engagement). In this research we have identified the five key elements of and relationships within such a DH ecosystem. We have established a solid foundation that can scope such an ecosystem from an Australian citizens’ perspective.

## Supporting information

S1 Appendix Data extraction and analysis. (DOCX)

S2 Appendix Studies included in the scoping review. (PDF)

S3 Appendix SCImago Journal Rank. (TIF)

S4 Appendix PRISMA-ScR checklist. (DOCX)
