# Supplementary material for: Understanding digital health ecosystem from Australian citizens’ perspective: A scoping review
Source: PLoS One. 2021 Nov 15;16(11):e0260058. doi: 10.1371/journal.pone.0260058 (PMC8592460; doi:10.1371/journal.pone.0260058)
Supplement: S1 Appendix — (DOCX) [file pone.0260058.s001.docx]

**S1 Appendix. Data Extraction and Analysis**

| **Reviewer: A** |  |  |  |  |  | |  |  |  | |  | |  |  |
| --- | --- | --- | --- | --- | --- | --- | --- | --- | --- | --- | --- | --- | --- | --- |
|  |  |  |  | **Classification (Yes / No / Unclear)** |  | |  |  | **Critical engagement** | | |  |  |  |
| **Title of paper** | **Authors** | **Year** | **Type of Study** | **Q1**  **What does the average Australian citizen know about DH? (Yes/No/Maybe)** | **Notes on Q1** | **Q2**  **What do they want from DH? (what are their needs and desires? (Yes/No/Maybe)** | | **Notes on Q2** | **Reviewer B**  **(Comment here)** | **Reviewer C**  **(Comment here)** | |  |  |  |
|  |  |  |  |  |  | |  |  |  |  | |  |  |  |
|  |  |  |  |  |  | |  |  |  |  | |  |  |  |
|  |  |  |  |  |  | |  |  |  |  | |  |  |  |
|  |  |  |  |  |  | |  |  |  |  | |  |  |  |
|  |  |  |  |  |  | |  |  |  |  | |  |  |  |
|  |  |  |  |  |  | |  |  |  |  | |  |  |  |
