# Supplementary material for: Understanding digital health ecosystem from Australian citizens’ perspective: A scoping review
Source: PLoS One. 2021 Nov 15;16(11):e0260058. doi: 10.1371/journal.pone.0260058 (PMC8592460; doi:10.1371/journal.pone.0260058)
Supplement: S2 Appendix — (PDF) [file pone.0260058.s002.pdf]

S2 Appendix. Studies included in the scoping review

Code	Paper	Authors	Year	Type of Study
1	Who Seeks Help Online for Self-Injury~	Mareka Frost and Leanne Casey	2016	Experimental Research Article
2	Characteristics of clients currently accessing a national online alcohol and drug counselling service	Garde, E. L., Manning, V., & Lubman, D. I.	2017	Experimental Research Article
3	eHealth System for Collecting and Utilizing Patient Reported Outcome Measures for Personalized Treatment and Care (PROMPT-Care) Among Cancer Patients~ Mixed Methods Approach to Evaluate Feasibility and Acceptability	Girgis, A., Durcinoska, I., Levesque, J., Gerges, M., Sandell, T., Arnold, A., & Delaney, G.	2017	Experimental Research Article
4	Is preference for mHealth intervention delivery platform associated with delivery platform familiarity~(Report)	Daniel Granger1, Corneel Vandelanotte1* Camille Short3 and Amanda Rebar	2016	Experimental Research Article
5	Use of the My Health Record by people with communication disability in Australia~ A review to inform the design and direction of future research	Hemsley, B., Georgiou, A., Carter, R., Hill, S., Higgins, I., van Vliet, P., & Balandin, S.	2016	Discussion / viewpoint
6	Cardiac Rehabilitation Online Pilot~ Extending Reach of Cardiac Rehabilitation	Higgins, O. R., Rogerson, M. M., Murphy, V. B., Navaratnam, C. H., Butler, C. M., Barker, C. L., . . . Jackson, C. A.	2017	Experimental Research Article
7	Consumer acceptance of patient-performed mobile teledermoscopy for the early detection of melanoma	Horsham, C., Loescher, L. J., Whiteman, D. C., Soyer, H. P., & Janda, M.	2016	Experimental Research Article
8	Be Positive Be Healthe~ Development and Implementation of a Targeted e-Health Weight Loss Program for Young Women	Hutchesson, M. J., Morgan, P. J., Callister, R., Pranata, I., Skinner, G., & Collins, C. E.	2016	Experimental Research Article
9	Exploring patient experiences and perspectives of a heart failure telerehabilitation program~ A mixed methods approach	Hwang, R., Mandrusiak, A., Morris, N. R., Peters, R., Korczyk, D., Bruning, J., & Russell, T.	2017	Experimental Research Article
10	Design Implications from the Preliminary Results of a Telemedicine Patient-Technology Interaction Study	Jalil, S., Myers, T., & Atkinson, I.	2014	Experimental Research Article
11	Diabetes Educators~ Perceived Experiences, Supports and Barriers to Use of Common Diabetes-Related Technologies	James, S., Perry, L., Gallagher, R., & Lowe, J.	2016	Experimental Research Article
12	Barriers for Delivering Telehealth in Rural Australia~ A Review Based on Australian Trials and Studies	Jang-Jaccard, J., Nepal, S., Alem, L., & Li, J.	2014	Review article
13	Investigating the preferences of older people for telehealth as a new model of health care service delivery~ A discrete choice experiment	Kaambwa, B., Ratcliffe, J., Shulver, W., Killington, M., Taylor, A., Crotty, M., . . . Kidd, M. R.	2017	Experimental Research Article
14	Online information and support needs of women with advanced breast cancer~ a qualitative analysis	Kemp, E., Koczwara, B., Butow, P., Turner, J., Girgis, A., Schofield, P., . . . Beatty, L.	2018	Experimental Research Article
15	Consumer Acceptance and Expectations of a Mobile Health Application to Photograph Skin Lesions for Early Detection of Melanoma	Koh, U., Horsham, C., Soyer, H. P., Loescher, L. J., Gillespie, N., Vagenas, D., & Janda, M.	2019	Experimental Research Article
16	Patient and practitioner satisfaction with tele-dermatology including Australia's indigenous population~ A systematic review of the literature	Kozera, E. K., Yang, A., & Murrell, D. F.	2016	Review article
17	Examining Internet and eHealth Practices and Preferences~ Survey Study of Australian Older Adults With Subjective Memory Complaints, Mild Cognitive Impairment, or Dementia	Lamonica, H. M., English, A., Hickie, I. B., Ip, J., Ireland, C., West, S., . . . Naismith, S. L.	2017	Experimental Research Article
18	Consumer Perceptions of and Willingness to Use Remotely Delivered Service Models For Exercise Management of Knee and Hip Osteoarthritis~ A Cross-Sectional Survey	BELINDA J. LAWFORD, KIM L. BENNELL, AND RANA S. HINMAN	2017	Experimental Research Article
19	Consumer Use of ~Dr Google~~ A Survey on Health Information-Seeking Behaviors and Navigational Needs	Lee, K., Hoti, K., Hughes, J., & Emmerton, L.	2015	Experimental Research Article
20	Healthdirect's After Hours GP Helpline-A Survey of Patient Satisfaction with the Service and Compliance with Advice	Li, L., Georgiou, A., Xiong, J., Byrne, M., Robinson, M., & Westbrook, J. I.	2016	Experimental Research Article
21	An 'integrated health neighbourhood'framework to optimise the use of EHR data	Liaw, S.-T., & de Lusignan, S.	2016	Discussion / viewpoint
22	Balancing self-tracking and surveillance~ legal, ethical and technological issues in using smartphones to monitor communication in people with health conditions.(Australia)	Jacki Liddle, Mark Burdon, David Ireland, Adrian Carter, Christina Knuepffer, Nastassja Milevskiy, Simon McBride, Helen Chenery and Wayne Hal	2016	Discussion / viewpoint
23	'Better understanding about what's going on'~ young Australians' use of digital technologies for health and fitness	Deborah Lupton	2018	Experimental Research Article
24	'It's made me a lot more aware'~ a new materialist analysis of health self-tracking	Deborah Lupton	2019	Experimental Research Article
25	Australian women's use of health and fitness apps and wearable devices~ a feminist new materialism analysis	Deborah Lupton	2019	Experimental Research Article
26	I'd like to think you could trust the government, but I don't really think we can'~ Australian women's attitudes to and experiences of My Health Record	Deborah Lupton	2019	Experimental Research Article
27	The more-than-human sensorium~ sensory engagements with digital self-tracking technologies	Deborah Lupton & Sarah Maslen	2018	Experimental Research Article
28	How Women Use Digital Technologies for Health~ Qualitative Interview and Focus Group Study	Deborah Lupton & Sarah Maslen	2019	Experimental Research Article
29	Attitudes Toward e-Mental Health Services in a Community Sample of Adults~ Online Survey	March, S., Day, J., Ritchie, G., Rowe, A., Gough, J., Hall, T., . . . Ireland, M.	2018	Experimental Research Article
30	Engaging consumers in their health data journey	Australia, C. H. F. o., & MedicineWise, N.	2018	Governmental Report
31	Beyond symptom monitoring~ Consumer needs for bipolar disorder self-management using smartphones	Nicholas, J., Boydell, K., & Christensen, H.	2017	Experimental Research Article
32	Systematic review of patient and caregivers' satisfaction with telehealth videoconferencing as a mode of service delivery in managing patients' health	Orlando, J., Beard, M., & Kumar, S.	2019	Review article
33	Citizens' use of digital media to connect with health care~ Socio-ethical and regulatory implications	Petersen, A., Tanner, C., & Munsie, M.	2019	Discussion / viewpoint
34	The influence of mobile health applications on patient - healthcare provider relationships~ A systematic, narrative review	Qudah, B., & Luetsch, K.	2019	Review article
35	mHealth applications as an educational and supportive resource for family carers of people with dementia~ An integrative review	Rathnayake, S., Moyle, W., Jones, C., & Calleja, P.	2019	Review article
36	End Users Want Alternative Intervention Delivery Models~ Usability and Acceptability of the REMOTE-CR Exercise-Based Cardiac Telerehabilitation Program	Rawstorn, J. C., Gant, N., Rolleston, A., Whittaker, R., Stewart, R., Benatar, J., . . . Maddison, R.	2018	Experimental Research Article
37	Telemedicine in the Northern Territory~ An assessment of patient perceptions in the preoperative anaesthetic clinic	Roberts, S., Spain, B., Hicks, C., London, J., & Tay, S.	2015	Experimental Research Article
38	Exploring the predictors of home telehealth uptake by elderly Australian healthcare consumers	Russell, T., Gillespie, N., Hartley, N., Theodoros, D., Hill, A., & Gray, L.	2015	Experimental Research Article
39	Telehealth~ experience of the first 120 consultations delivered from a new Refugee Telehealth clinic	Schulz, T. R., Richards, M., Gasko, H., Lohrey, J., Hibbert, M. E., & Biggs, B. A.	2014	Experimental Research Article
40	E-Health readiness in outback communities~ an exploratory study	Schwarz, F., Ward, J., & Willcock, S.	2014	Experimental Research Article
41	Ethical Implications of User Perceptions of Wearable Devices	Segura Anaya, L., Alsadoon, A., Costadopoulos, N., & Prasad, P.	2018	Experimental Research Article
42	Consumer preferences for teledermoscopy screening to detect melanoma early	Spinks, J., Janda, M., Soyer, H. P., & Whitty, J. A.	2016	Experimental Research Article
43	Flying blind~ Australian consumers and digital health	Srinivasan, U., Rao, S., Ramachandran, D., & Jonas, D.	2016	Governmental Report
44	Highly digital consumers are more skeptical of healthcare transparency services	Stephan, J.-P., Kalis, B., Brombach, M., & Nickell, J.	2015	White paper
45	Consumer expectations and healthcare in Australia	Taylor, M., & Hill, S.	2014	White paper
46	Are people with severe mental illness ready for online interventions~ Access and use of the Internet in Australian mental health service users	Thomas, N., Foley, F., Lindblom, K., & Lee, S.	2017	Experimental Research Article
47	Demographic characteristics of Australian health consumers who were early registrants for opt-in personally controlled electronic health records	Emma Torrens and Sue M walker	2017	Experimental Research Article
48	The Use of Mobile Applications Among Adults with Type 1 and Type 2 Diabetes~ Results from the Second MILES—Australia (MILES-2) Study	Trawley, S., Baptista, S., Browne, J. L., Pouwer, F., & Speight, J.	2017	Experimental Research Article

49	Examining user perceptions of SwallowIT~ A pilot study of a new telepractice application for delivering intensive swallowing therapy to head and neck cancer patients	Wall, L. R., Ward, E. C., Cartmill, B., Hill, A. J., & Porceddu, S. V.	2017	Experimental Research Article
50	A content analysis of the consumer-facing online information about My Health Record~ Implications for increasing knowledge and awareness to facilitate uptake and use	Walsh, L., Hill, S., Allan, M., Balandin, S., Georgiou, A., Higgins, I., . . . Hemsley, B.	2018	Review article
51	Enablers and barriers in providing telediabetes services for Indigenous communities~ A systematic review	Wickramasinghe, S. I., Caffery, L. J., Bradford, N. K., & Smith, A. C.	2016	Review article
52	Patient centred systems~ Techno-anthropological reflections on the challenges of 'meaningfully engaging' patients within health informatics research	Wong, M. C., Almond, H., Cummings, E., Roehrer, E., Showell, C., & Turner, P.	2015	Discussion / viewpoint
53	Understanding individual users’ perspectives on the personally controlled electronic health record (PCEHR) system~ Results of field study	Xu, J., Gao, X., Hammond, J., Antonius, N., & Sorwar, G.	2018	Experimental Research Article
54	Using diffusion of innovation theory to understand the factors impacting patient acceptance and use of consumer e-health innovations~ a case study in a primary care clinic	Zhang, X., Yu, P., Yan, J., & Ton A M Spil, I.	2015	Experimental Research Article
55	Users’ preferences and design recommendations to promote engagements with mobile apps for diabetes selfmanagement: Multi-national perspectives	Adu, M. D., Malabu, U. H., Malau-Aduli, A. E. O., & Malau-Aduli, B. S.	2018	Experimental Research Article
56	Safety concerns with consumer-facing mobile health applications and their consequences: a scoping review	Akbar, S., Coiera, E., & Magrabi, F.	2019	Review article
57	Determinants of access to eHealth services in regional Australia	Alam, K., Mahumud, R. A., Alam, F., Keramat, S. A., Erdiaw-Kwasie, M. O., & Sarker, A. R	2019	Experimental Research Article
58	‘Recognition of competition’ versus Will to App: rethinking digital engagement in Australian youth sexual health promotion policy and practice	Kath Albury	2019	Discussion / viewpoint
59	Prejudices and perceptions: patient acceptance of mobile	Alexander, S. M., Nerminathan, A., Harrison, A., Phelps, M., & Scott, K. M.	2015	Experimental Research Article
60	Recommendations for enhancing the implementation and utility of shared digital health records in rural Australian communities	Almond, H., Cummings, E., & Turner, P.	2018	Experimental Research Article
61	Mobile Health Apps to Facilitate Self-Care: A Qualitative Study of User Experiences	Kevin Anderson, Oksana Burford, Lynne Emmerton	2016	Experimental Research Article
62	The Australian general public’s perceptions of having a personally controlled electronic health record (PCEHR)	Andrews, L., Gajanayake, R., & Sahama, T.	2014	Experimental Research Article
63	Current patient and healthcare worker attitudes to eHealth and the personally controlled electronic health record in major hospitals	Armani, R., Mitchell, L. E., Allen-Graham, J., Heriot, N. R., Kotsimbos, T., & Wilson, J. W.	2016	Experimental Research Article
64	Consumer acceptance of Accountable-eHealth systems	Gajanayake, R., Iannella, R., & Sahama, T. R.	2014	Experimental Research Article
65	A National Patient-Centered E-Health Solution–The Experience from Down Under To-Date with the Personally Controlled Electronic Health Record (PCEHR)	Muhammad, I., & Wickramasinghe, N.	2014	Discussion / viewpoint
66	Evaluation of a National Broadband Network-enabled Telehealth trial for older people with chronic disease	Nancarrow, S., Banbury, A., & Buckley, J.	2016	Experimental Research Article
67	End-user acceptance of a cloud-based teledentistry system and Android phone app for remote screening for oral diseases	Mohamed Estai1, Yogesan Kanagasingam2, Di Xiao2, Janardhan Vignarajan2, Stuart Bunt1, Estie Kruger1 and Marc Tennant	2017	Experimental Research Article
68	Maternity Patients' Access to Their Electronic Medical Records~ Use and Perspectives of a Patient Portal	Megan Forster, Kerrie Dennison, Joanne Callen, Andrew Georgiou and Johanna I. Westbrook	2015	Experimental Research Article
69	Patient perspectives on a personally controlled electronic health record used in regional Australia~ ‘I can be like my own doctor’	Hanna, L., Gill, S. D., Newstead, L., Hawkins, M., & Osborne, R. H.	2017	Experimental Research Article
70	Exploring the use of technology pathways to access health information by Australian university students: a multi-dimensional approach	Usher, W., Gudes, O., & Parekh, S.	2016	Experimental Research Article
71	The medium, the message and the measure: a theory-driven review on the value of telehealth as a patient-facing digital health innovation	Abimbola, S., Keelan, S., Everett, M., Casburn, K., Mitchell, M., Burchfield, K., & Martiniuk, A.	2019	Review article
72	Elements of Trust in Digital Health Systems: Scoping Review	Adjekum, A., Blasimme, A., & Vayena, E.	2018	Review article
73	Transforming digital health services in Australia	Agnola, D.	2018	Discussion / viewpoint
74	Preferences for Online Mental Health Services Among Australian and Indian Samples: A Cross-Cultural Comparison	Austin, D. W., Bhola, P., Tebble, C., & Shandley, K.	2018	Experimental Research Article
75	A comparison of characteristics of patients seen in a tertiary hospital diabetes telehealth service versus specialist face-to-face outpatients	Menon, A., Gray, L. C., Fatehi, F., Darssan, D., Bird, D., Bennetts, D., & Russell, A. W.	2017	Experimental Research Article
76	Multi-site videoconferencing for home-based education of older people with chronic conditions: the Telehealth Literacy Project	Banbury, A., Parkinson, L., Nancarrow, S., Dart, J., Gray, L., & Buckley, J.	2014	Experimental Research Article
77	What Do Adults with Type 2 Diabetes Want from the ‘‘Perfect’’ App? Results from the Second Diabetes MILES: Australia (MILES-2) Study	Baptista, S., Trawley, S., Pouwer, F., Oldenburg, B., Wadley, G., & Speight, J.	2019	Experimental Research Article
78	Evaluating the prevalence and opportunity for technology use in chronic kidney disease patients: a cross-sectional study	Bonner, A., Gillespie, K., Campbell, K. L., Corones-Watkins, K., Hayes, B., Harvie, B., . . . Havas, K.	2018	Experimental Research Article
79	Consumer Health-Related Activities on Social Media: Exploratory Study	Bpharm, A., Chen, T., & Aslani, P.	2017	Experimental Research Article
80	Awareness, experiences and perceptions of telehealth in a rural Queensland community	Bradford, N., Caffery, L., & Smith, A.	2015	Experimental Research Article
81	Nutrition Interventions for Prevention and Management of Childhood Obesity: What Do Parents Want from an eHealth Program?	Burrows, T., Hutchesson, M., Chai, L., Rollo, M., Skinner, G., & Collins, C.	2015	Experimental Research Article
82	How telehealth facilitates the provision of culturally appropriate healthcare for Indigenous Australians	Caffery, L. J., Bradford, N. K., Smith, A. C., & Langbecker, D.	2018	Experimental Research Article
83	Outcomes of using telehealth for the provision of healthcare to Aboriginal and Torres Strait Islander people: a systematic review	Caffery, L. J., Bradford, N. K., Wickramasinghe, S. I., Hayman, N., & Smith, A. C.	2017	Review article
84	Evaluation research studies essential to ensuring health information systems meet the needs of users, including patients	Joanne Callen	2016	Discussion / viewpoint
85	Implementation factors are neglected in research investigating telehealth delivery of allied health services to rural children: A scoping review	Campbell, J., Theodoros, D., Hartley, N., Russell, T., & Gillespie, N.	2019	Review article
86	Will Patients Accept Daily SMS as a Communication to Support Adherence to Mental Health Treatment	Bonnie A Clough and Leanne M Casey	2018	Experimental Research Article
87	Going global~ do consumer preferences, attitudes, and barriers to using e-mental health services differ across countries~	Bonnie A. Clough, Mostafa Zarean, Ilse Ruane, Niño Jose Mateo, Turana A. Aliyeva & Leanne M. Casey	2017	Experimental Research Article
88	eHealth for inflammatory bowel disease self-management - the patient perspective	Danny Con, Belinda Jackson, Kathleen Gray & Peter De Cruz	2017	Experimental Research Article
89	Patients are willing to use telehealth for the multidisciplinary management of chronic musculoskeletal conditions~ A cross-sectional survey	Michelle A Cottrell, Anne J Hill, Shaun P O’Leary, Maree E Raymer and Trevor G Russell	2018	Experimental Research Article
90	Looking to tomorrow’s healthcare today~ a participatory health perspective	Sheryl Coughlin, David Roberts, Kenneth O’Neill, Peter Brooks<br>David Roberts,2 Kenneth O’Neill3 and Peter Brooks	2018	Discussion / viewpoint
91	Feasibility and Acceptability of Remotely Monitored Pedometer-Guided Physical Activity	J. N. Darvall, A. Parker, D. A. Story	2016	Experimental Research Article
92	Patient Portals Facilitating Engagement With Inpatient Electronic Medical Records~ A Systematic Review	Ronald Dendere1*, PhD; Christine Slade2*, PhD; Andrew Burton-Jones3*, PhD; Clair Sullivan1,4*, MBBS (Hons), MD, FACHI; Andrew Staib5,6*, MBBS, FACHI; Monika Janda	2019	Review article
93	Going Online~ The Affordances of Online Counseling for Families Affected by Alcohol and Other Drug Issues	Ella Dilkes-Frayne1,2 , Michael Savic1,3, Adrian Carter1,4, Renata Kokanović5	2019	Experimental Research Article
94	User Experience of an Innovative Mobile Health Program to Assist in Insulin Dose Adjustment~ Outcomes of a Proof-Of-Concept Trial	Ding, H., Fatehi, F., Russell, A. W., Karunanithi, M., Menon, A., Bird, D., & Gray, L. C.	2018	Experimental Research Article
95	Older Patients' Perspectives of Online Health Approaches in Chronic Obstructive Pulmonary Disease	Disler, R. T., Inglis, S. C., Newton, P., Currow, D. C., Macdonald, P. S., Glanville, A. R., . . . Davidson, P. M.	2019	Experimental Research Article

96	Where else would i look for it~ A five-country qualitative study on purposes, strategies, and consequences of online health information seeking	Diviani, N., Fredriksen, E. H., Meppelink, C. S., Mullan, J., Rich, W., & Sudmann, T. T.	2019	Experimental Research Article
97	Availability, spatial accessibility, utilisation and the role of telehealth for multi-disciplinary paediatric cerebral palsy services in Queensland	Sisira Edirippulige1, John Reyno1, Nigel R Armfield1,2, Matthew Bambling3, Owen Lloyd4 and Elizabeth McNevin	2016	Experimental Research Article
98	Patient access to electronic health records~ Differences across ten countries	Essén, A., Scandurra, I., Gerrits, R., Humphrey, G., Johansen, M. A., Kierkegaard, P., . . . Ancker, J. S.	2018	Review article
