# Supplementary figures and images for: Understanding digital health ecosystem from Australian citizens’ perspective: A scoping review
Source: PLoS One. 2021 Nov 15;16(11):e0260058. doi: 10.1371/journal.pone.0260058 (PMC8592460; doi:10.1371/journal.pone.0260058)

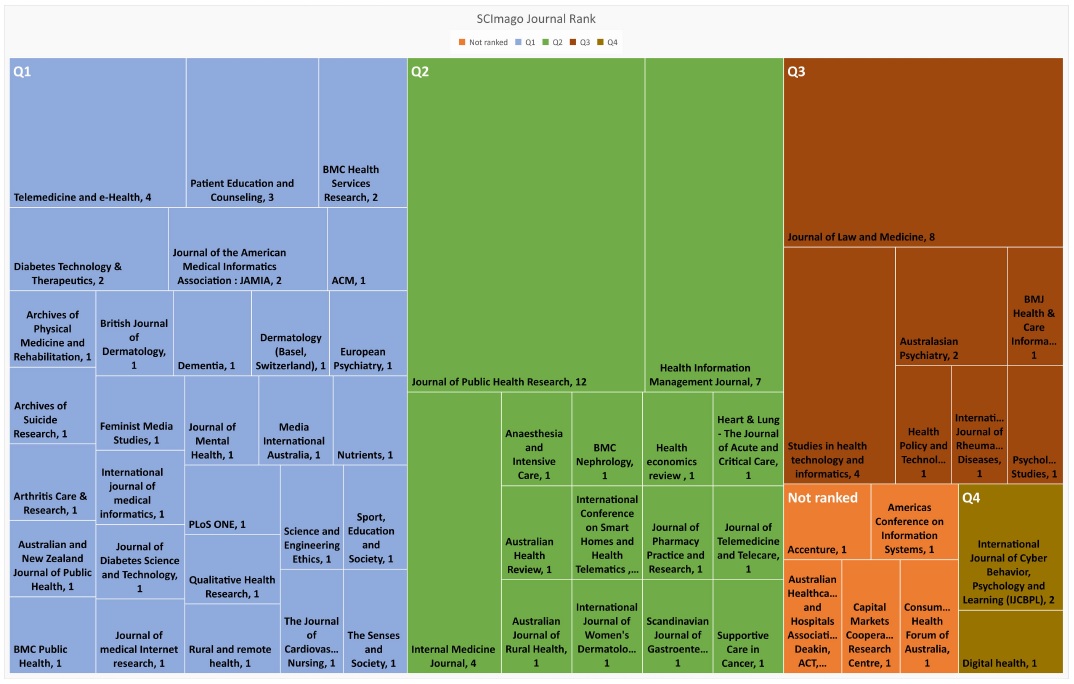

Supplement: S3 Appendix — (TIF) [file pone.0260058.s003.tif]
